# Choroidal Changes During and After Discontinuing Long-Term 0.01% Atropine Treatment for Myopia Control

**DOI:** 10.1167/iovs.65.10.21

**Published:** 2024-08-13

**Authors:** Samantha Sze-Yee Lee, Gareth Lingham, Antony Clark, Scott A. Read, David Alonso-Caneiro, David A. Mackey

**Affiliations:** 1 University of Western Australia, Centre for Ophthalmology and Visual Science (incorporating the Lions Eye Institute), Perth, Western Australia, Australia; 2 Centre for Eye Research Ireland, Environmental, Sustainability and Health Institute, Technological University Dublin, Dublin, Ireland; 3 Perth Children's Hospital, Perth, Western Australia, Australia; 4 Queensland University of Technology, Contact Lens and Visual Optics Laboratory, Centre for Vision and Eye Research, Optometry and Vision Science, Kelvin Grove, Queensland, Australia; 5 School of Science, Technology, and Engineering, University of Sunshine Coast, Petrie, Queensland, Australia; 6 School of Medicine, Menzies Research Institute Tasmania, University of Tasmania, Hobart, Tasmania, Australia; 7 Centre for Eye Research Australia, University of Melbourne, Royal Victorian Eye and Ear Hospital, Melbourne, Victoria, Australia

**Keywords:** atropine, australia, choroidal thickness (ChT), choroidal vascularity index (CVI), myopia control, randomized controlled trial

## Abstract

**Purpose:**

Few studies have explored choroidal changes after cessation of myopia control. This study evaluated the choroidal thickness (ChT) and choroidal vascularity index (CVI) during and after discontinuing long-term low-concentration atropine eye drops use for myopia control.

**Methods:**

Children with progressive myopia (6–16 years; *n* = 153) were randomized to receive 0.01% atropine eye drops or a placebo (2:1 ratio) instilled daily over 2 years, followed by a 1-year washout (no eye drop use). Optical coherence tomography imaging of the choroid was conducted at the baseline, 2-year (end of treatment phase), and 3-year (end of washout phase) visits. The main outcome measure was the subfoveal ChT. Secondary measures include the CVI.

**Results:**

During the treatment phase, the subfoveal choroids in both treatment and control groups thickened by 12–14 µm (group difference *P* = 0.56). During the washout phase, the subfoveal choroids in the placebo group continued to thicken by 6.6 µm (95% confidence interval [CI] = 1.7 to 11.6), but those in the atropine group did not change (estimate = -0.04 µm; 95% CI = –3.2 to 3.1). Participants with good axial eye growth control had greater choroidal thickening than the fast-progressors during the treatment phase regardless of the treatment group (*P* < 0.001), but choroidal thickening in the atropine group's fast-progressors was not sustained after stopping eye drops. CVI decreased in both groups during the treatment phase, but increased in the placebo group after treatment cessation.

**Conclusions:**

On average, compared to placebo, 0.01% atropine eye drop treatment did not cause a differential rate of change in ChT during treatment, but abrupt cessation of long-term 0.01% atropine eye drops may disrupt normal choroidal thickening in children.

An inverse association between choroidal thickness (ChT) and refractive error, as quantified by spherical equivalent (SphE) is well-documented, with myopic and longer eyes typically having thinner choroids.^1^^–^^4^ The choroid is also believed to play a role in the visually guided regulation of eye growth^3^^,^^4^ by undergoing rapid changes in response to retinal defocus, moving the retina forward or backward such that the image focuses on the retina.^5^^–^^7^ Studies have consistently shown that short-term myopic defocus induces choroidal thickening, whereas hyperopic defocus — a myopigenic stimulus — results in choroidal thinning in humans regardless of age.^5^^–^^7^

ChT has therefore been suggested to be a biomarker for refractive error^1^^,^^8^ and, more recently, as an indicator of the effectiveness of myopia control.^9^^–^^11^ For example, instillation of atropine eye drops, an effective myopia control treatment, leads to a rapid thickening of the choroid.^4^^,^^12^ Recently, the low-concentration atropine for myopia progression (LAMP) study^9^ reported that daily instillation of 0.05% or 0.025% atropine eye drops resulted in thickening of the subfoveal choroid after 4 months, which was sustained for the remainder of the 2-year trial period. The authors also reported that up to 18.5% atropine eye drops’ anti-myopia effect was mediated via changes in the ChT. Importantly, low-concentration atropine eye drops have been shown to counter the choroidal thinning effects induced by myopia-inducing stimuli, such as hyperopic defocus,^13^^,^^14^ providing insights to the drug's anti-myopigenic mechanism.

Cessation of atropine eye drops after long-term use for myopia control has been associated with a “rebound” effect, where the rate of myopia progression increases after stopping eye drops compared to during, and even before, atropine use.^15^^–^^17^ However, changes in ChT and any associations with rebound myopia progression after long-term atropine treatment is discontinued are largely unexplored.

Changes to the choroid induced by atropine eye drops could also potentially affect choroidal vasculature. Xu et al.^18^ reported that the choroidal vascularity index (CVI; which is the ratio of the vascular luminal area to the total choroidal area within a cross-sectional scan of the choroid) significantly decreased during the first 6 months of daily 0.01% atropine instillation, whereas no significant changes in CVI were noted with weekly 1% atropine use. Like ChT, changes to the CVI or its related measures following atropine cessation have not been evaluated.

Recently, we reported the 2-year treatment^19^ and third-year washout^17^ results of our Western Australia atropine for the treatment of myopia (WA-ATOM) study. Our trial found that 0.01% atropine eye drops had a moderate effect on slowing myopia progression up to 18 months, with waning effects between 18 and 24 months.^19^ Following eye drop cessation in the third year, rebound was noted in the treatment group such that the cumulative myopia progression was similar between the treatment and control groups by the end of the 3-year study.^17^ The current analysis aims to investigate the changes in choroidal measures and their associations with myopia progression over the course of the treatment and washout phases of the WA-ATOM study.

## Methods

This analysis was conducted as part of our double-blind randomized controlled trial that compared 0.01% atropine eye drops to a placebo for myopia control in a multi-racial cohort of Australian children. The full methodology^20^ and myopia control outcomes^17^^,^^19^ of the trial have been described previously. In brief, 153 children (6–16 years old) with myopia (measured as SphE) of –1.50 diopters (D) or worse, documented myopia progression of ≥0.50 D in the 12 months prior to the index date, astigmatism ≤1.50 D, and anisometropia ≤1.00 D were randomized to receive 0.01% atropine eye drops or a placebo at a 2:1 ratio. Children with connective tissue disorders, ocular pathology, amblyopia, or binocular vision abnormalities apart from heterophoria were excluded. The study comprised a 2-year treatment phase where participants were instructed to instill eye drops on a nightly basis, followed by a 1-year washout phase where all eye drop use ceased. Participants and study investigators remained masked to the treatment allocation throughout the 3 years.

At enrollment, children and their parents or caregivers were given a full explanation of the nature of the study, after which the children gave their verbal assent while their parents or caregivers provided written consent for their child to participate. This trial was approved by the University of Western Australia Human Research Ethics Committee and was conducted in accordance with the Declaration of Helsinki. This trial was registered in the Australia and New Zealand Clinical Trials Registry (ACTRN12617000598381). The use of the 0.01% atropine eye drops and placebo were approved by the Therapeutics Goods Administration, Department of Health, Australia.

### Eye Examination and Optical Coherence Tomography Imaging

Participants’ axial length (IOLMaster V5; Carl Zeiss Meditec AG, Jena, Germany) and refractive error (Nidek ARK-510A autorefractometer; NIDEK Co. Ltd., Japan) were measured every 6 months throughout the 3-year trial. Autorefraction was performed at least 20 minutes after 1 to 3 drops of 1% cyclopentolate instillation, with cycloplegia being confirmed through assessment of the light pupil response.

At 0, 24, and 36 months, which correspond to the visits at baseline, end of the treatment phase, and end of the washout phase, participants underwent spectral domain optical coherence tomography (SD-OCT; Spectralis HRA + OCT; Heidelberg Engineering, Heidelberg, Germany) chorioretinal imaging of the macula, with an axial and transverse resolution of 3.9 and 5.6 µm, respectively.

Prior to SD-OCT imaging, participants wore their habitual optical correction to undergo testing of their distance and near visual acuity, accommodative amplitude, and stereoacuity. Spectacles were then removed for pupillometry, ocular biometry, tonometry, and cycloplegic eye drop instillation. All tests except for pupillometry was done in a well-lit room (250–500 lux). Participants were not given any special instruction to avoid any activity that may affect choroidal thickness, such as exercise or near work, prior to the appointment.

**Figure 1.**
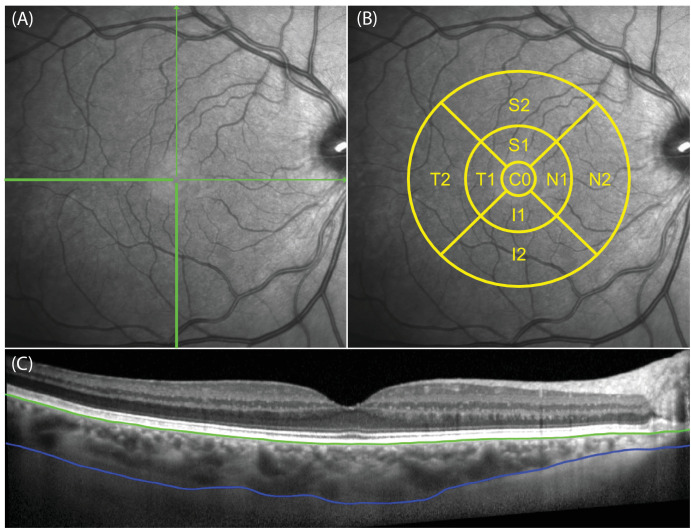
Choroidal thickness imaging and measurement. (**A**) Horizontal and vertical 30 degrees B-scans centered on the fovea; (**B**) thickness were estimated at each subfield of the early treatment of diabetic retinopathy grid which comprises a central 0.5mm radius circle centered on the fovea (C0), and the superior (S), inferior (I), temporal (T), and nasal (N) segments of the inner (between 0.5 and 1.5 mm radii circles) and outer (between 1.5 and 3.0 mm radii circles) rings; (**C**) machine-learning program segmentation of the retinal pigmented epithelium (*green line*) and chorioscleral interface (*blue line*).

SD-OCT imaging was done in a room with lights at 250 to 300 lux. Corneal curvature for each eye was entered into the SD-OCT device to correct for magnification effects. The Enhance Depth Imaging (EDI) mode was implemented and 2 horizontal and 2 vertical 30 degrees B-scans centered on the fovea were obtained (Fig. 1A), with an average of 100 frames for each scan recorded. Scans with a signal-to-noise ratio of <20 were discarded. Scans taken at baseline were set as the reference image on which the 24- and 36-month imagings were based.

### Choroid Image Analyses

The EDI images were exported from the SD-OCT and analyzed using non-commercial custom deep-learning software written on MATLAB (Mathworks, Inc., Natick, MA, USA), as described previously.^21^^–^^23^ First, the choroid was delineated in each of the B-scans by the software (Fig. 1C). This segmentation was checked by an experienced observer masked to the treatment status of the participants, and corrected manually if required. Next, the ChT was measured at 10 µm intervals across each B-scan. We further accounted for transverse magnification effects due to variations in axial length between eyes using a customized MATLAB script. From these corrected measurements, the ChT within each subfield of the Early Treatment for Diabetic Retinopathy (ETDRS) grid was calculated (Fig. 1B).

**Figure 2.**
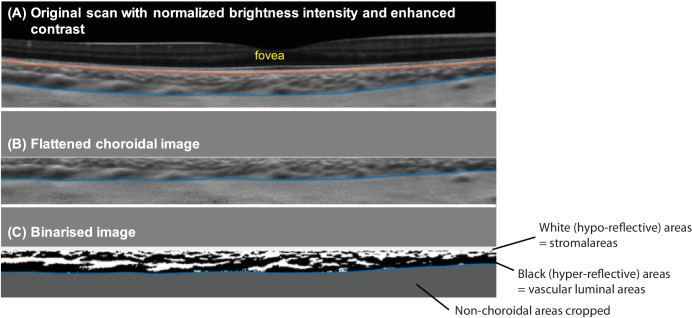
Binarization of cross-sectional choroidal scans. (**A**) The original grey-scale scan was manipulated such that (**B**) the naturally curved choroid is flattened to (**C**) allow binarization, whereas the non-choroidal areas were cropped out. The white and black areas of the binarized images corresponds to the stromal and luminal areas, respectively. The choroidal vascularity index is the ratio of the luminal area to the total choroidal area within the cross-sectional scan.

To obtain the CVI, the segmented images of the cross-sectional choroidal scans underwent binarization, which converted the grey-scale images into black and white, corresponding to the vascular luminal and stromal areas, respectively (Fig. 2), using a previously validated methed.^23^ The method first flattened the choroid region using the retinal pigment layer as a reference, then fed the image into a trained deep-learning model to quantify choroidal vascularity (see Fig. 2).^23^ The CVI was calculated as the percentage of the cross-sectional thickness of the choroid taken up by the vascular luminal thickness (see Fig. 2), with greater CVI representing a higher proportion of blood vessels within the measured area of the choroid. Additionally, the luminal thickness was computed as CVI × ChT, whereas the stromal thickness was computed as (1 − CVI) × ChT. The CVI, luminal thickness, and stromal thicknesses within the subfoveal choroid (central 0.5 mm radius) were derived.^23^

The primary choroidal outcome measure was the subfoveal (central 0.5 mm radius circle subfield) ChT. Secondary outcome measures are the ChT at the other eight ETDRS subfields and the choroidal vascularity measures – CVI, luminal thickness, and stromal thickness – subfoveally. Participants without choroidal data at the baseline visit or with low-quality/truncated scans were excluded.

### Statistical Analysis

Analyses were conducted on an intention-to-treat basis using R version 4.1.1 (2021 The R Foundation for Statistical Computing Platform [https://www.r-project.org/]), with the level of significance set at *P* < 0.05. Linear mixed-effect models were used to explore the longitudinal changes in choroidal measures against treatment group, as well as myopia progression against choroidal measures and treatment group. Linear mixed-effect models were chosen due to the robustness against missing data, with comparable performance with multiple imputation.^24^ To compare choroidal changes between participants with fast and slow progression (whether with 0.01% atropine or placebo) in terms of slowing eye growth, we conducted additional subgroup analyses. “Slow progressors” were determined as those with axial elongation less than or equal to the sample median during the 2-year treatment phase, whereas the remaining eyes were classified as “fast progressors.” A random intercept term was entered into each model to account for the repeated measures within participants (2 eyes and multiple visits). All analyses controlled for age and baseline value of the outcome measure. Given the diurnal variations in ChT and that participants were seen at various times in the day, for analyses with choroidal measures as the outcome, we additionally controlled for time of day of SD-OCT imaging in the statistical models.

## Results

**Figure 3.**
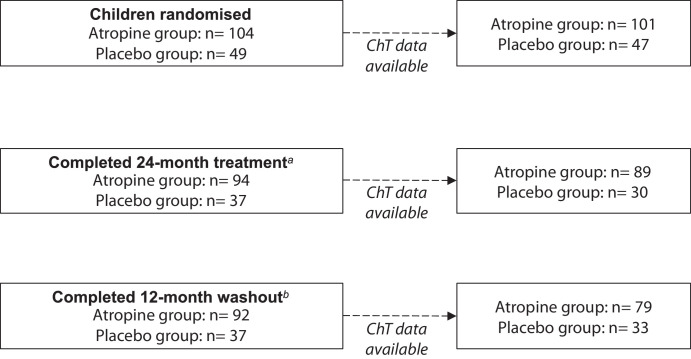
Sample size and retention. ^a^Reasons for withdrawal: Three did not like diagnostic or study drops; four had difficulty adhering to the treatment regimen; three had difficulty attending appointments + concern of receiving placebo; two were uncontactable; one relocated; three wanted to seek myopia treatment (atropine or orthokeratology) privately; two were uncontactable; and four cited personal reasons/did not provide reason. ^b^Reasons for withdrawal: One sought atropine and orthokeratology eye drops privately, and one was uncontactable.

Of the 153 children enrolled, 3 participants in the treatment group and 2 in the placebo group were excluded due to truncated scans or inadequate imaging quality at baseline, leaving 148 children (96.7%) in the analysis. At the 24- and 36-month visits, 124 (81.0%) and 120 (78.4%) children had ChT data available for analysis (Fig. 3). There was no difference in baseline age, sex, ethnicity, allocated treatment group, or other baseline measures between those included in and those excluded from the analysis (*P* ≥ 0.16).

Participant demography and baseline measures are shown in Table 1. Median ChTs at each ETDRS subfield according to treatment group and visit are shown in Supplementary Figure S1. The placebo group were, on average, approximately 1 year older than those in the atropine group, whereas sex, ethnicity, and eye measures did not significantly differ between groups.

**Table 1.** Demography and Myopia Measures at Baseline of Included Sample

	Placebo (*n* = 47)	0.01% Atropine (*n* = 101)	*P* Value^*^
Index age, y; mean ± SD	12.2 ± 2.5	11.2 ± 2.7	0.034^*^
Female sex	28 (59.6%)	58 (57.4%)	0.95
Ethnicity			
Europeans	21 (44.7%)	47 (46.5%)	0.88
East Asians	9 (19.1%)	18 (17.8%)	
South Asians	11 (23.4%)	22 (21.8%)	
Other	6 (12.8%)	14 (13.9%)	
*Baseline ocular measures*			
*SphE progression rate prior to study, D/y; median [IQR]*	−1.00 [−1.27 to 0.69]	−0.95 [−1.27 to 0.62]	0.98
SphE, D; median [IQR]	−3.50 [−4.50 to −2.63]	−3.13 [−4.13 to −2.38]	0.29
AL, mm; median [IQR]	24.8 [24.3 to 25.4]	24.6 [24.2 to 25.2]	0.21
Subfoveal ChT, µm; median [IQR]	256.1 [229.5 to 303.4]	252.4 [212.2 to 296.1]	0.33
Subfoveal CVI, %; mean ± SD	61.0 [58.1 to 65.3]	61.4 [58.8 to 66.5]	0.27
Subfoveal luminal thickness, µm; mean ± SD	156.0 [142.7 to 179.3]	157.1 [137.4 to 185.1]	0.77
Subfoveal stromal thickness, µm; mean ± SD	94.5 [81.4 to 117.6]	93.0 [75.3 to 119.7]	0.34

AL = axial length; ChT = choroidal thickness; CVI = choroidal vascularity index; D = diopters; IQR = interquartile range; SD = standard deviation; SphE = spherical equivalent.

* Difference between groups analyzed using independent t-test for index age, chi-square test for sex and ethnicity, and linear mixed effect model for ocular measures, significantly different between groups at *P* < 0.05.

### Choroidal Thickness Changes

**Figure 4.**
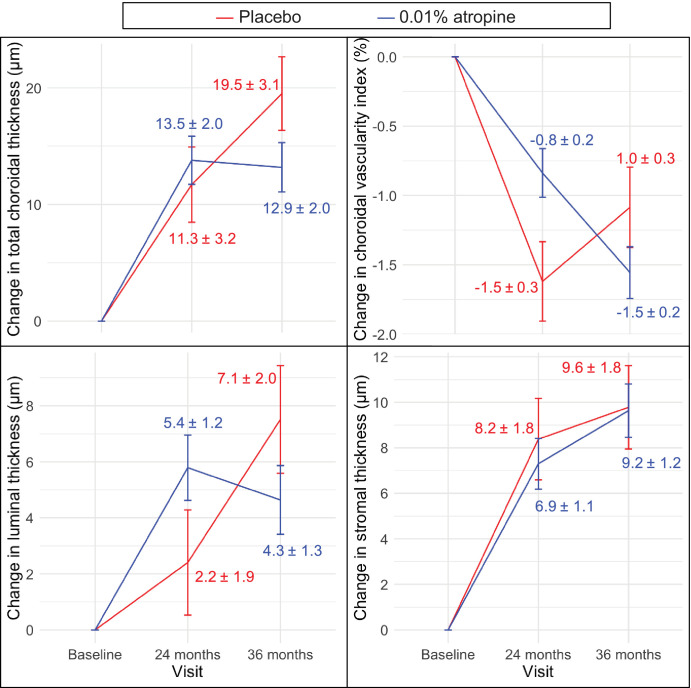
Figure numbers indicate the estimated marginal mean change in subfoveal choroidal measures from baseline. Error bars represent standard errors.

**Figure 5.**
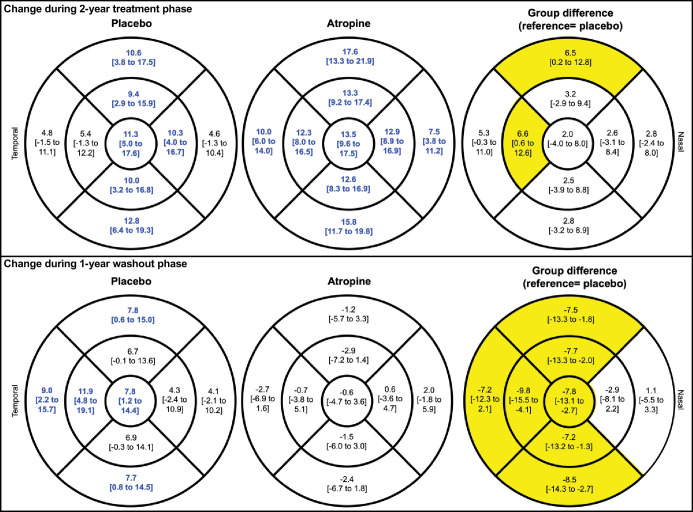
Estimated marginal mean change in choroidal thickness (µm) and 95% confidence interval during the 2-year treatment phase (*top panel*) and the 1-year washout phase (*bottom panel*). The placebo and atropine groups are shown in the *left and middle columns*, respectively. *Blue numbers* indicate significant thickening of the choroid during either phase at *P* < 0.05. Group difference in the amount of change is shown in the *right column*. The *yellow grids* indicate significant difference between groups at *P* < 0.05. Figures corrected for age, baseline value, and time of imaging.

Figure 4 and Table 2 show the estimated marginal mean change in subfoveal ChT. The estimated marginal mean change in ChT in all subfields are shown in Figure 5, in addition to the raw descriptive change shown in Supplementary Table S1. We examined the time of choroidal imaging and found no statistical difference between the placebo and treatment groups at the baseline (*P* = 0.84), 24-month (*P* = 0.30), or 36-month (*P* = 0.66) visits. At the end of the treatment phase, the atropine-treated group had significantly thicker choroids in all subfields of the ETDRS compared to baseline (see Fig. 5). The placebo group similarly had an increase in ChT in all but the temporal and outer nasal subfields. In all subfields, the choroidal thickening was greater in the atropine group than the placebo, although this difference was statistically significant only at the inner temporal and outer superior regions (*P* = 0.040 and 0.049, respectively; see Fig. 5).

**Table 2.** Estimated Marginal Means Change^*^ (and 95% CI) in Subfoveal Choroidal Measures and Myopia Outcomes

	Change During 2-Y Treatment Phase			Change During 1-Y Washout Phase		
	Placebo Group	0.01% Atropine Group	*P* Value	Placebo Group	0.01% Atropine Group	*P* Value
Choroidal measures						
Full thickness, µm	+11.3	+13.5	0.46	−7.0	−1.2	0.004^‡^
	(95% CI = 5.0 to 17.6)	(95% CI = 9.6 to 17.4)		(95% CI = 1.5 to 12.4)	(95% CI = −4.8 to 2.3)	
CVI, %	−1.5	−0.8	0.001^‡^	+0.2	−0.8	<0.001^‡^
	(95% CI = −2.1 to −0.9)	(95% CI = −1.2 to −0.4)		(95% CI = −0.4 to 0.7)	(95% CI = −1.1 to −0.4)	
Luminal thickness, µm	+2.2	+5.4	0.11	+3.6	−1.1	0.008^‡^
	(95% CI = −1.5 to 5.9)	(95% CI = 3.0 to 7.8)		(95% CI = 0.2 to 7.0)	(95% CI = −3.2 to 1.0)	
Stromal thickness, µm	+8.2	+6.9	0.40	+1.9	+2.7	0.67
	(95% CI = 4.7 to 11.7)	(95% CI = 4.7 to 9.1)		(95% CI = −1.3 to 5.1)	(95% CI = 0.7 to 4.7)	
Myopia outcomes						
Spherical equivalent, D	−0.74	−0.66	0.58	−0.28	−0.39	0.029^†^
	(95% CI = −0.86 to −0.62)	(95% CI = −0.73 to −0.59)		(95% CI = −0.36 to −0.20)	(95% CI = −0.44 to −0.34)	
Axial length, mm	0.35	0.34	0.66	0.12	0.19	<0.001^‡^
	(95% CI = 0.30 to 0.40)	(95% CI = 0.31 to 0.37)		(95% CI = 0.09 to 0.14)	(95% CI = 0.17 to 0.21)	

* Radius 0.5 µm centered on fovea; adjusted for age and baseline value.

Group difference significantly different at ^†^*P* < 0.05 and ^‡^*P* < 0.01.

CI = confidence interval; CVI = choroidal vascularity index; SE = standard error.

During the washout phase, the placebo group continued to have a thickening of the choroid at several regions (see Fig. 5, Supplementary Table S1). The subfoveal (*P* = 0.009), inner temporal (*P* = 0.002), outer temporal (*P* = 0.019), and outer inferior subfields (*P* = 0.016) were significantly thicker at 36 months compared to 24 months (see Fig. 5). On the other hand, the atropine group exhibited a trend toward a thinning of the choroid in most subfields of the ETDRS grid, although none of these were statistically significant (see Fig. 5). The change in ChT during the washout phase was significantly different between groups at the subfoveal (*P* = 0.004), and all other regions (*P* ≤ 0.018) except for the nasal subfields.

### Choroidal Vascularity Changes

As shown in Figure 4 and Supplementary Table S1, by the end of 2 years of treatment, the CVI decreased in both groups (both *P* < 0.001) with a greater reduction in the placebo group (estimate = 1.5%, 95% confidence interval [CI] = 1.0 to 2.1) than the treatment group (estimate = 0.8%, 95% CI = 0.5 to 1.1). During the 1-year washout, the CVI in the atropine group decreases by a further 0.7% (95% CI = 0.3 to 1.1, *P* < 0.001), but the change in the placebo group was not significant (estimate = 0.5%, 95% CI = –0.1 to 1.1, *P* = 0.11).

Luminal thickness increased in both groups during the treatment phase (see Fig. 4), but this increase was significant only in the atropine group (estimate = 5.4 µm, 95% CI = 3.1 to 7.8, *P* < 0.001). During the 1-year washout, however, the luminal thickness in the atropine group did not significantly change (*P* = 0.37), whereas the placebo group had a significant thickening of the luminal layer by 5.1 µm (95% CI = 1.0 to 9.2, *P* = 0.016. This group difference in the change in luminal thickness during both phases were significant (treatment phase *P* = 0.042 and washout phase *P* = 0.011).

Across both groups, the stromal thickness increased by an average of 8.2 µm (95% CI = 4.7 to 11.7) by the end of the treatment phase. The change in stromal thickness during the washout phase was not significant (estimate = 1.4 µm; 95% CI = –1.4 to 5.5; see Fig. 4). The amount of change between the placebo and control groups did not differ significantly (group difference *P* = 0.53 during the treatment phase and *P* = 0.69 during the washout phase).

### Choroidal Thickness and Myopia Progression

The change in SphE and axial length in both phases are shown in Table 3. The cumulative myopia progression and axial elongation from baseline to the end of the washout phase was not significantly different between groups (*P* ≥ 0.58).

**Table 3.** Change in Rate of Myopia Progression (Estimate [95% CI]) Against Subfoveal Choroidal Measures^*^

	2-Y Treatment			1-Y Washout		
	All Participants (Main Effect)	Placebo Group	Atropine Group	All Participants (Main Effect)	Placebo Group	Atropine Group
Per 10 µm increase in choroidal thickness						
Change in SphE	0.06^‡^	0.05^§^	0.12^§^	0.02	−0.13	0.02^†^
	[0.02 to 0.09]	[0.02 to 0.07]	[0.10 to 0.14]	[−0.02 to 0.05]	[−0.53 to 0.23]	[0.00 to 0.034]
Change in AL	−0.04^§^	−0.03^§^	−0.05^§^	−0.02^§^	−0.02^§^	−0.01^‡^
	[−0.05 to −0.02]	[−0.05 to −0.02]	[−0.06 to −0.04]	[−0.03 to −0.01]	[−0.03 to −0.01]	[−0.02 to −0.00]
Per 10% increase in choroidal vascularity index						
Change in SphE	−0.45	0.10	−0.02	−0.075	0.13	0.03
	[−0.99 to 0.07]	[−0.27 to 0.45]	[−0.53 to 0.13]	[−0.33 to 0.44]	[−0.53 to 0.23]	[−0.23 to 0.17]
Change in AL	0.03	0.02	0.53	0.09	0.10	0.02
	[−0.15 to 0.22]	[−0.12 to 0.16]	[0.07 to 0.15]	[−0.04 to 0.19]	[−0.03 to 0.20]	[−0.04 to 0.07]
Per 10 µm increase in luminal thickness (µm)						
Change in SphE	0.11^§^	0.09^‡^	0.11^§^	0.01	0.02	0.03
	[−0.65 to 4.28]	[0.03 to 0.14]	[0.06 to 0.16]	[−0.07 to 0.09]	[−0.06 to 0.08]	[−0.00 to 0.07]
Change in AL	−0.04^§^	−0.03^‡^	−0.05^§^	−0.00	−0.00	−0.01
	[−0.05 to 0.03]	[−0.06 to −0.00]	[−0.06 to −0.03]	[−0.04 to 0.01]	[−0.02 to −0.02]	[−0.02 to 0.00]
Per 10 µm increase in stromal thickness (µm)						
Change in SphE	0.09^§^	0.08^†^	0.09^§^	0.04	0.05	0.01
	[0.05 to 0.14]	[0.01 to 0.16]	[0.03 to 0.14]	[−0.04 to 0.13]	[−0.05 to 0.16]	[−0.01 to 0.05]
Change in AL	−0.073^‡^	−0.03^†^	−0.04^§^	−0.00	−0.03	−0.00
	[−0.05 to −0.02]	[−0.08 to −0.00]	[−0.05 to −0.02]	[−0.02 to 0.00]	[−0.05 to 0.00]	[−0.01 to 0.00]

AL = axial length; ChT = choroidal thickness; CI = confidence interval; SphE = spherical equivalent.

* Adjusted for age, baseline value, and time of choroidal thickness imaging. Statistically significant change at ^†^*P* < 0.05, ^‡^*P* < 0.01, or ^§^*P* < 0.001.

**Figure 6.**
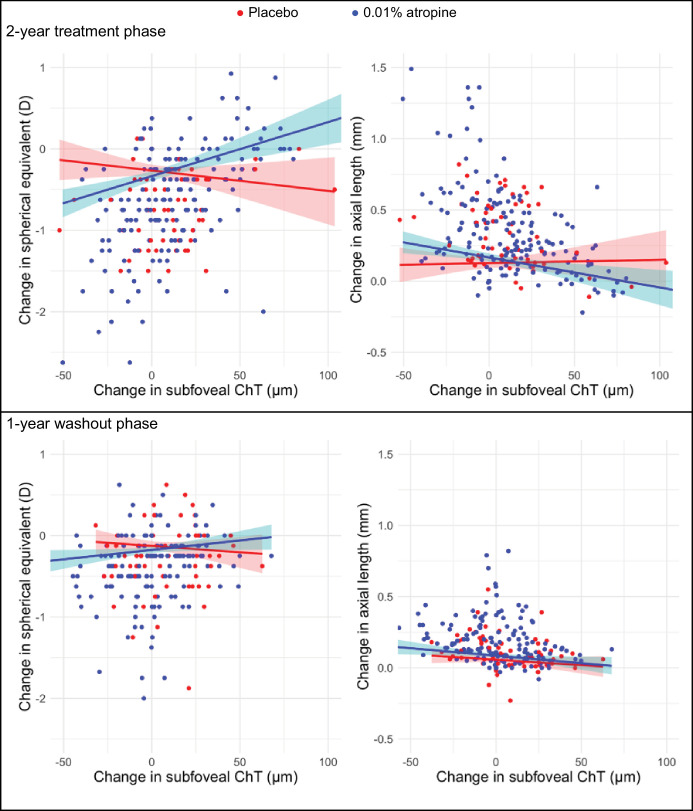
Myopia progression against change in subfoveal choroidal thickness (ChT) during treatment phase (*top panel*) and washout phase (*bottom panel*).

During the treatment phase, greater thickening of the subfoveal choroids was associated with slower myopia progression in both groups (Table 3). There was additionally a group × ChT interaction effect on changes in the SphE and axial length (*P* = 0.001 and 0.037), such that the relationship between subfoveal ChT change and myopia progression was stronger in the atropine than in the placebo group (Fig. 6). During the washout phase, ChT did not have a main effect on SphE, but there was a significant group × ChT interaction effect on SphE change, such that thicker ChT was associated with slower SphE change only in the atropine group, but not the placebo group (see Fig. 6, Table 3). There was a main effect of ChT change on axial elongation (*P* = 0.006), but the ChT change × treatment group interaction effect on axial elongation was not statistically significant. A mediation analysis did not reveal any effect of atropine eye drops mediated via the ChT on myopia progression.

### Choroidal Vascularity and Myopia Progression

There was no main effect of CVI change on myopia progression during either phase (see Table 3). A greater increase in luminal or stromal thickness was significantly associated with reduced myopia progression, in terms of both SphE and axial length change, during the treatment phase. These associations were not significant during the washout, nor was there a differential effect of luminal thickness on myopia progression between groups (see Table 3). Mediation analyses showed no effect of treatment exerted via changes in choroidal vascularity measures on myopia progression.

### Choroidal Measures and Axial Elongation

Across the entire sample, axial length increased by a median of 0.27 mm during the treatment phase, unadjusted for other factors. Eyes with axial elongation ≤0.27 mm during the 2 years were thus categorized as “slow progressors” (*n* = 91 eyes in the atropine group and *n* = 31 eyes in the placebo group), and the remaining as “fast progressors” (*n* = 109 in the atropine group and *n* = 62 eyes in the placebo group). The slow progressors were older on average than those with fast progression (mean age = 12.6 ± 2.3 and 10.4 ± 2.4 years, respectively; difference *P* < 0.001).

**Figure 7.**
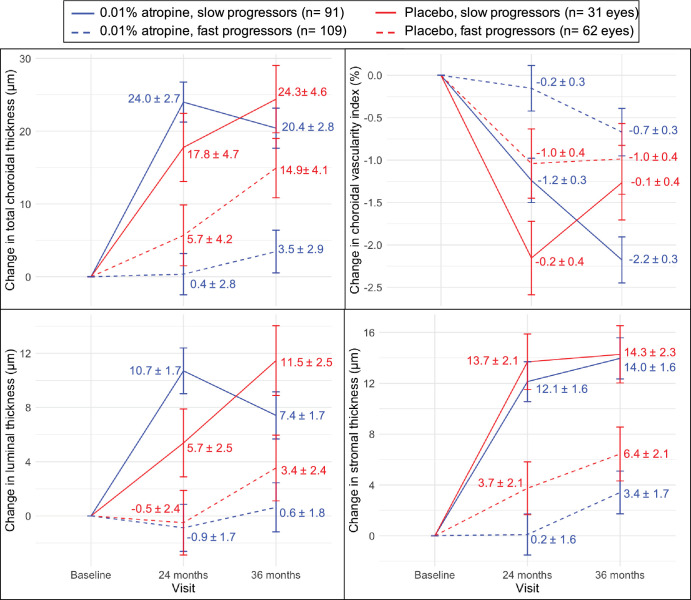
Figure numbers indicate the estimated marginal mean change (±1 standard error) change in subfoveal choroidal measures from baseline according to treatment group and axial elongation.

The raw changes in choroidal measures in both groups are shown in Supplementary Table S2. Over the 2-year treatment phase across both treatment groups, the slow progressors had greater thickening in total choroidal, luminal, and stromal layers, as well as greater decrease in CVI compared to those with faster progression (Fig. 7, Table 4), after controlling for age and baseline ChT. There was a treatment group × progression subgroup interaction effect on the thickness measures, such that the differences in the amount of choroidal thickening between the slow and fast progressors were greater within the atropine group than the placebo group (see Fig. 7, Table 4). During the washout phase, none of the choroidal measures changed significantly, regardless of treatment group or progression subgroup, nor was there any treatment group by progression interaction effect (see Table 4).

**Table 4.** Difference in Change in Subfoveal^*^ Choroidal Measures Based on Axial Elongation (Reference = Fast Progressors)^†^

	Main Effect (Across Both Groups)	Effect in Placebo Group Only	Effect in 0.01% Atropine Group Only	Interaction Effect^‡^
Effect during 2-y treatment phase^§^				
Choroidal thickness, µm	23.5 (95% CI = 15.4 to 31.9)^#^	17.4 (95% CI = 2.0 to 32.7)	25.9 (95% CI = −35.3 to −16.4)^#^	*P* < 0.001^#^
CVI, %	−0.9 (95% CI = −1.9 to −0.0)^||^	−1.6 (95% CI = −3.1 to 0.1)	−0.8 (95% CI = −1.8 to 0.4)	*P* = 0.06
Luminal thickness, µm	12.5 (95% CI = 7.6 to 17.5)^#^	7.6 (95% CI = −1.6 to 16.9)	14.1 (95% CI = 8.3 to 19.9)*^#^*	*P* < 0.001^#^
Stromal thickness, µm	12.5 (95% CI = 7.6 to 17.5)^#^	12.7 (95% CI = 3.6 to 21.7)*^||^*	13.2 (95% CI = 7.5 to 18.9)*^#^*	*P* < 0.001^#^
Effect during 1-y washout phase^§^				
Choroidal thickness	−0.2 (95% CI = −8.1 to 7.6)	5.1 (95% CI = −8.9 to 19.0)	−0.7 (95% CI = −9.9 to 8.5)	*P* = 0.88
CVI, %	−0.5 (95% CI = −1.3 to 0.3)	0.2 (95% CI = −1.3 to 1.8)	−0.5 (95% CI = −1.4 to 0.4)	*P* = 0.29
Luminal thickness, µm	−1.2 (95% CI = −6.2 to 3.8)	1.6 (95% CI = −8.2 to 11.5)	−1.3 (95% CI = −7.0 to 4.4)	*P* = 0.66
Stromal thickness, µm	0.4 (95% CI = −4.1 to 4.9)	−0.3 (95% CI = −8.8 to 8.2)	0.5 (95% CI = −4.6 to 5.7)	*P* = 0.83

* Subfoveal = 0.5 mm radius around fovea center.

† Slow progressors defined as eyes with axial elongation no more than the sample median of 0.27 mm during the 2-y treatment phase while the remaining categorized as fast progressors.

‡ Treatment group (placebo versus 0.01% atropine) by axial elongation (slow vs fast progression) interaction effect.

§ Controlled for age and baseline value.

CVI = choroidal vascularity index. Statistically significant at ^||^ *P* < 0.5 and ^#^ *P* < 0.001, adjusted for age, baseline values, and time of imaging.

## Discussion

During normal childhood eye growth, there is typically a thickening of the choroid which continues into young adulthood,^1^^,^^2^^,^^25^^,^^26^ although there have been reports of choroidal thinning during childhood in children with myopic shifts^27^^,^^28^ or in those of Asian descent.^29^^–^^31^ This choroidal thickening has been suggested to serve as a mechanism to slow eye growth during ocular development.^3^ In our current multiethnic cohort, on average, there was a thickening of the choroid over the first 2 years of the study, regardless of whether the children were receiving 0.01% atropine eye drops or a placebo.

Our findings are at odds with those of the LAMP study,^9^ which found no significant change in ChT, and even a trend toward a thinning of the choroid, after 2 years of daily 0.01% atropine or placebo eye drop instillation. The contrast in findings is likely related to the difference in the amount of myopia progression between our study populations. Although both our and the LAMP studies included children with progressive myopia, during the first 12 months of our respective studies, the placebo and 0.01% atropine groups in the LAMP study had, on average, 0.41 and 0.36 mm in axial elongation, respectively, compared to only 0.25 and 0.16 mm in our WA-ATOM study.

The age difference between studies may be another reason for the difference in findings. A cross-sectional study^32^ found that the choroid was thicker in girls who are in later pubertal stages compared to age-matched controls, although no such relationships were found in boys. Our older cohort is likely to comprise more children in the later stages of puberty, and thus possibly increased choroidal thickening, as opposed to the LAMP study. Whereas both groups had thicker choroids by the end of the treatment phase relative to baseline, after stopping eye drops, this thickening continued in the placebo group, but not in the atropine group. Although the atropine group comprise younger children who were expected to have faster myopia progression, and thus less choroidal thinning, younger age is also associated with faster choroidal thickening.^29^ Instead, we found limited choroidal thickening in the younger treatment group during the washout phase. Although we have controlled for age in our analyses, this age difference between groups may have resulted in an underestimation of the group difference during the washout phase of the study.

The pattern of change in total ChT throughout the 3 years was somewhat mirrored by the changes in the choroidal luminal thickness, although the atropine group had a significantly greater thickening by the end of the treatment phase compared to the placebo group. The changes in stromal thickness, on the other hand, had the same amount of increase in both the atropine and placebo groups throughout the 3 years. This suggests that the differential changes in the total ChT between groups were mainly driven by the luminal thickness changes in this cohort, and that abrupt cessation of long-term low-concentration atropine eye drop use leads to reduced thickening of the vascular luminal layer of the choroid. The changes in luminal thickness in the treatment group suggest a potential change in blood flow associated with cessation of atropine. This will need to be confirmed with future work using functional choroidal imaging, such as OCT-angiography.

Importantly, regardless of whether children were receiving 0.01% atropine or placebo, those with good axial length control (i.e. slower eye growth) had thicker choroids by the end of the treatment phase, whereas minimal changes in the ChT were noted in those with more rapid axial elongation. This difference in choroidal change was noted in spite of the age difference. The choroid tends to thicken during normal childhood growth, with a faster rate of thickening occurring earlier in childhood.^29^ Thus, the younger age of those with faster progression would be expected to show a greater thickening of their choroids. Instead, the choroidal thickness remained largely unchanged in the subgroup with faster progression. It is hence likely that the difference between progression subgroup has been underestimated in the current study due to the age difference.

Interestingly, in the atropine group, the choroidal thickening in the slow progressors did not continue after stopping eye drops, as opposed to the placebo group in whom the choroid continued to thicken regardless of progression subgroup. This pause in choroidal thickening after atropine cessation corresponds to the rebound myopia progression noted in this group.

Two important inferences can be made from these above observations. First, any choroidal thickening associated with atropine eye drops is not sustained in the long term after eye drop cessation. This is supported by an 8-week study by Jiang et al.^33^ who reported an increase in subfoveal ChT from a baseline of 280 to 307 µm by the end of 1 week of a loading dose of atropine (1% atropine administered twice a day), which then reverted to its baseline thickness 7 weeks after atropine use ceased.

Second, abrupt cessation of long-term low-concentration atropine eye drops may hinder normal choroidal thickening. As discussed briefly, choroidal thickening during childhood may serve to control ocular axial growth.^3^ The halt in normal choroidal thickening after cessation of atropine eye drops may predispose to or indicate rapid myopia progression. Indeed, the pause in choroidal thickening in the atropine group during the washout phase corresponds to their faster decline in SphE.

Despite the similar amount of choroidal thickening during the treatment phase in both groups, the inverse relationship between change in ChT and amount of myopia progression was significantly stronger in the atropine group. Moreover, choroidal thickening was greater in those in the atropine group with good axial elongation control compared to those in the placebo group with similar axial elongation. This suggests that the possible protective effect of choroidal thickening may be enhanced with atropine eye drops.

Over the 3 years, we further observed a decrease in CVI. Even though the luminal thickness generally increased over the study period, there was an even greater increase in stromal thickness which drove the reduction in CVI. Although CVI changes were not significantly associated with myopia progression, thickening of either the luminal and stromal layers were linked with slower SphE and axial length change during the 2-year treatment period. However, we failed to find a significant mediation effect of any of the choroidal measures on SphE or axial length change. This may be due to our small sample size and the weak effect of 0.01% atropine on myopia control.

The inclusion of a placebo-control group in the current study is a main strength of the current findings, as opposed to other mid- or long-term studies,^12^^,^^14^^,^^34^ including the LAMP study^9^ in which a placebo-control group crossed over to receive 0.05% atropine mid-way into the treatment phase. Our placebo-control group allowed us to profile the natural history of ChT changes against which the intervention group could be compared in parallel.

However, our study may be limited by the measurement of ChT only at 3 time points – baseline, 24 months, and 36 months. We were thus unable to confirm the linearity of ChT changes between these follow ups. Nonetheless, the current findings suggest that abrupt cessation of 0.01% atropine eye drops result in a long-term reduction in normal choroidal thickening during childhood. Further experiments with more frequent choroidal imaging to explore the patterns of ChT changes during long-term atropine use and after cessation are required to ascertain the impact of this myopia treatment on choroidal outcomes.

Another potential limitation of the current study is the impact of diurnal variation on the measured ChT. Participants were examined at various times of the day to allow them to attend study visits at their convenience. Although we have accounted for time of SD-OCT imaging in the statistical analysis, we acknowledge that it may not fully negate the effects of diurnal changes in the ChT.

Our study demonstrated that after long-term 0.01% atropine eye drop use is discontinued, normal choroidal thickening in children is disrupted, suggesting that this may underlie the rebound myopia progression following eye drop cessation. The longer-term outcome of stopping atropine eye drops on the ChT and any relationship with myopia progression should be investigated in future studies. Such studies may provide further insights into the underlying mechanism of atropine's anti-myopigenic effects and the role of the choroid in myopia progression.

## Supplementary Material

Supplement 1
