# Supplementary material for: Choroidal Changes During and After Discontinuing Long-Term 0.01% Atropine Treatment for Myopia Control
Source: Invest Ophthalmol Vis Sci. 2024 Aug 13;65(10):21. doi: 10.1167/iovs.65.10.21 (PMC11323994; doi:10.1167/iovs.65.10.21)
Supplement: Supplement 1 [file iovs-65-10-21_s001.pdf]

Supplementary Table 1. Descriptive statistics (median and IQR) of raw (unadjusted) change in choroidal measures

	Change during 2-year treatment phase		Change during 1-year washout phase	
	**Placebo group**	**0.01% atropine group**	**Placebo group**	**0.01% atropine group**
Choroidal thickness (μm)				
Subfoveal	10.8 [IQR= -3.3 to 22.6]	9.9 [IQR= -7.2 to 30.4]	3.9 [IQR= -7.7 to 24.6]	-0.1 [IQR= -14.5 to 15.4]
Inner superior	9.5 [IQR= -6.2 to 19.3]	8.7 [IQR= -8.5 to 29.1]	2.8 [IQR= -10.2 to 20.5]	-0.2 [IQR= -15.2 to 13.3]
Inner inferior	7.3 [IQR= -3.3 to 22.1]	9.9 [IQR= -8.5 to 28.6]	5.5 [IQR= -11.3 to 24.6]	-0.1 [IQR= -13.8 to 17.2]
Inner temporal	3.0 [IQR= -9.2 to 15.9]	10.1 [IQR= -8.3 to 28.3]	8.5 [IQR= -6.9 to 24.7]	-0.4 [IQR= -14.0 to 14.6]
Inner nasal	9.2 [IQR= -2.9 to 22.6]	10.0 [IQR= -6.6 to 28.4]	5.9 [IQR= -8.8 to 18.3]	0.9 [IQR= -13.2 to 16.5]
Outer superior	9.0 [IQR= -4.1 to 22.9]	13.3 [IQR= -1.3 to 33.0]	5.7 [IQR= -11.8 to 18.3]	0.3 [IQR= -15.3 to 13.6]
Outer inferior	11.3 [IQR= 0.0 to 25.6]	11.0 [IQR= -5.1 to 34.7]	5.2 [IQR= -9.0 to 19.4]	1.0 [IQR= -13.6 to 14.2]
Outer temporal	5.5 [IQR= -6.3 to 17.9]	7.9 [IQR= -8.0 to 24.8]	9.0 [IQR= -6.9 to 16.2]	-0.1 [IQR= -12.6 to 11.6]
Outer nasal	3.1 [IQR= -8.6 to 17.5]	6.7 [IQR= -6.5 to 25.5]	5.0 [IQR= -3.6 to 15.7]	2.3 [IQR= -10.5 to 15.1]
Vascularity measures at subfoveal choroid				
CVI (%)	-1.2 [IQR= -2.4 to 0.4]	-0.6 [IQR= -2.3 to 0.7]	-0.2 [IQR= -1.7 to 1.2]	-0.5 [IQR= -1.7 to 0.4]
Luminal thickness (μm)	1.4 [IQR= -6.9 to 12.3]	3.7 [IQR= -4.1 to 14.4]	2.5 [IQR= -6.8 to 12.5]	-0.2 [IQR= -10.0 to 8.8]
Stromal thickness (μm)	5.6 [IQR= -2.3 to 15.9]	3.9 [IQR= -4.4 to 14.7]	3.3 [IQR= -5.7 to 10.2]	2.3 [IQR= -4.6 to 9.9]

Subfoveal = 0.5mm radius; inner = between 0.5 and 1.5 mm radius; outer= between 1.5 and 3.0 mm radius. CVI= choroidal vascularity index; IQR= interquartile range.

Supplementary Table 2. Descriptive statistics (median and IQR) of raw (unadjusted) change in subfoveal[a] choroidal measures between those with good and poor responses[b]

Treatment group	Placebo group		0.01% atropine group	
**Response**	**Good (n= 31 eyes)**	**Poor (n= 62 eyes)**	**Good (n= 91 eyes)**	**Poor (n= 109 eyes)**
Change during 2-year treatment phase				
Choroidal thickness (µm)	15.4 [-3.3 to 26.9]	8.3 [-3.3 to 16.4]	20.1 [2.6 no 45.3]	0.0 [-13.3 to 13.8]
CVI (%)	-1.4 [-2.5 to -0.1]	-0.6 [-2.3 to 0.8]	-1.3 [-2.5 to 0.3]	-0.1 [-1.6 to 1.2]
Luminal thickness (µm)	1.9 [-5.1 to 23.8]	0.8 [-8.8 to 9.2]	8.4 [-0.1 to 23.9]	-0.5 [-7.0 to 5.9]
Stromal thickness (µm)	14.0 [1.5 to 23.8]	2.5 [-2.8 to 8.5]	10.2 [0.7 to 21.3]	-0.3 [-6.9 to 6.8]
Change during 1-year washout phase				
Choroidal thickness	0.1 [-8.6 to 24.9]	5.5 [-1.0 to 23.5]	-2.9 [-21.1 to 17.9]	1.4 [-8.8 to 13.6]
CVI (%)	-0.2 [-1.7 to 9.1]	-0.1 [-1.5 to 2.1]	-0.6 [-2.0 to 0.6]	-0.3 [-1.3 to 0.3]
Luminal thickness (µm)	2.8 [-6.8 to 12.6]	2.2 [-1.9 to 11.2]	-0.8 [-13.4 to 9.1]	1.3 [-5.1 to 8.6]
Stromal thickness (µm)	3.7 [-8.6 to 24.9]	1.7 [-9.3 to 7.5]	1.4 [-5.6 to 12.3]	3.3 [-2.9 to 8.1]

[a] Subfoveal= 0.5mm radius around fovea center; [b] good responders defined as eyes with axial elongation no more than the sample median of 0.27mm during the 2-year treatment phase while the remaining categorized as poor response. CVI= choroidal vascularity index
